# Important role of kallikrein 6 for the development of keratinocyte proliferative resistance to topical glucocorticoids

**DOI:** 10.18632/oncotarget.9926

**Published:** 2016-06-08

**Authors:** Mari Kishibe, Gleb Baida, Pankaj Bhalla, Robert M. Lavker, Bethanee Schlosser, Sin Iinuma, Shigetaka Yoshida, Joel T. Dudley, Irina Budunova

**Affiliations:** ^1^ Department Dermatology, Asahikawa Medical University, Japan; ^2^ Department Dermatology, Northwestern University, Chicago, IL, USA; ^3^ Department Functional Anatomy and Neuroscience, Asahikawa Medical University, Japan; ^4^ Department Genetics and Genomic Sciences, Icahn School of Medicine at Mount Sinai, New York, NY, USA

**Keywords:** glucocorticoid, glucocorticoid receptor, skin atrophy, tachyphylaxis, kallikrein 6

## Abstract

One of the major adverse effects of topical glucocorticoids is cutaneous atrophy often followed by development of resistance to steroids (tachyphylaxis). Previously we showed that after two weeks, interfollicular mouse keratinocytes acquired resistance to anti-proliferative effects of glucocorticoid fluocinolone acetonide (FA). One of the top genes activated by FA during tachyphylaxis was *Klk6* encoding kallikrein-related peptidase 6, known to enhance keratinocyte proliferation. *KLK6* was also strongly induced by chronic glucocorticoids in human skin. Double immunostaining showed that KLK6^+^ keratinocytes, localized in suprabasal layer of mouse skin, were frequently adjacent to proliferating 5-bromo-2'-deoxyuridine-positive basal keratinocytes. We used KLK6 knockout (KO) mice to evaluate KLK6 role in skin regeneration after steroid-induced atrophy. KLK6 KOs had thinner epidermis and decreased keratinocyte proliferation. The keratinocytes in wild type and KLK6 KO epidermis were equally sensitive to acute anti-proliferative effect of FA. However, the development of proliferative resistance during chronic treatment was reduced in KO epidermis. This was not due to the changes in glucocorticoid receptor (GR) expression or function as GR protein level and induction of GR-target genes were similar in wild type and KLK6 KO skin. Overall, these results suggest a novel mechanism of epidermal regeneration after glucocorticoid-induced atrophy via KLK6 activation.

## INTRODUCTION

Glucocorticoids have potent anti-inflammatory and anti-proliferative activity, and are the most frequently prescribed drugs for the treatment of various skin diseases including psoriasis and atopic dermatitis [1,2]. However, chronic use of topical corticosteroids is associated with adverse effects, the major one being skin atrophy. Steroid-induced skin atrophy involves epidermis, dermis, epidermal appendages and subcutaneous fat [1,3–6]. It is characterized by epidermal thinning, decreased number and size of keratinocytes, diminished stratum corneum and intercellular lipid lamella, which together with atrophic changes in other skin compartments ultimately result in decreased skin barrier function.

Along with the development of skin atrophy, keratinocytes, vascular and lymphoid cells in the skin develop resistance to glucocorticoids (tachyphylaxis) [7–9]. We and others previously showed that after two weeks of topical steroid treatment, interfollicular keratinocytes in mouse skin lost sensitivity to glucocorticoid-induced anti-proliferative effects, and that keratinocyte proliferation that was drastically reduced at the beginning of treatment (10-15% of control level) returned to, or exceeded, the control level later after chronic treatment [10,11]. As discussed below, steroid tachyphylaxis in patients is also well documented, even though in some cases it may reflect irregular steroid use [8,9,12–14].

The effects of glucocorticoids are mediated by the glucocorticoid receptor (GR), a well-characterized transcription factor [15,16]. In non-stimulated cells, GR resides in the cytoplasm. Upon hormone binding, GR translocates to the nucleus where it regulates the expression of target genes via binding to glucocorticoid-responsive elements (GRE) in their promoters/enhancers or via other mechanisms including tethering interaction with other transcription factors at protein level [15,16].

We recently discovered that glucocorticoids induced a robust activation of kallikrein-related peptidase 6 (KLK6), in mouse and human skin ([17] and our DNA array, GEO submission number GSE59151). KLK6 (also called protease M/neurosin/serine protease 9 or 19/zyme) is a secreted trypsin-like serine proteinase from the kallikrein family [18]. KLK6 was discovered and cloned by several independent laboratories and later was reported to be highly expressed during inflammation, as well as in many cancers and cancer cell lines [18–20].

In the skin, KLK6 is weakly expressed in the differentiated layers (strata corneum and granulosum) of the epidermis and in skin appendages: sebaceous and eccrine sweat glands [21,22]. Along with several other KLKs, KLK6 is able to cleave the corneocyte adhesion molecule, desmoglein 1 [22]. KLK6 and other KLKs have been suggested to lead to the cleavage of other desmosomal proteins either directly or through participation in a proteolytic activation cascade and thus, contribute to the shedding of dead corneocytes in the process of epidermal desquamation [22,23].

In addition, KLK6 regulates keratinocyte proliferation and migration. Ectopic KLK6 overexpression in a mouse keratinocyte cell line, induced a spindle-type morphology, accelerated cell growth, and enhanced migration and invasiveness [21]. It also led to increased ectodomain shedding of E-cadherin resulting in reduced cell-cell adhesion [21].

Corresponding to its roles in epidermal proliferation and desquamation, KLK6 is overexpressed in skin of patients with hyperproliferative/hyperkeratotic/inflammatory skin diseases including psoriasis, atopic dermatitis and peeling skin syndrome-type B, as well as in premalignant skin lesions and squamous cell carcinomas [21,24–27]. Similarly, in mice, KLK6 expression is strongly activated in skin by the irritant/tumor promoter, 12-*O*-tetradecanoylphorbol 13-acetate, which induces both keratinocyte proliferation and differentiation [23].

All previously published data suggested that KLK6 can counteract the anti-proliferative pressure of glucocorticoids and may play an important role in epidermal regeneration after steroid-induced skin atrophy. The goals of the current study were to assess the expression of KLK6 at mRNA/protein levels during glucocorticoid-induced skin atrophy and to determine its role in the development of resistance to the glucocorticoids using KLK6 knockout (KO) mice [28].

We report here that chronic topical treatment with glucocorticoids induced KLK6 expression in murine and human skin. In mice, KLK6 was induced in suprabasal keratinocytes of the interfollicular epidermis (IFE) often adjacent to 5-bromo-2'-deoxyuridine (BrdU)^+^ proliferating keratinocytes, suggesting an important role in the induction of proliferation. Furthermore, we found that KLK6 KO animals had a thinner epidermis and decreased keratinocyte proliferation. Even though the interfollicular keratinocytes in KLK6 KO and wild type (WT) mice were similarly sensitive to growth inhibition after a single dose of glucocorticoid fluocinolone acetonide (FA), the development of keratinocyte proliferative resistance to FA was significantly reduced in KLK6 KO mice.

## RESULTS

### KLK6 expression is induced in mouse and human skin during chronic glucocorticoid treatment

**Figure 1:**
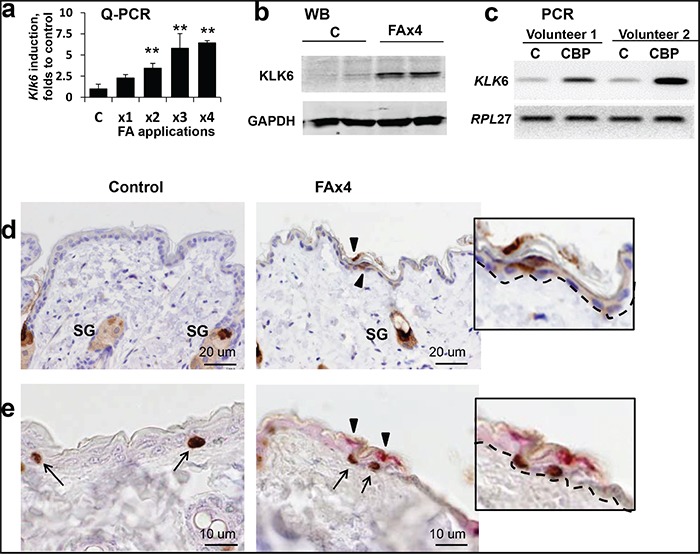
Induction of KLK6 expression in skin after chronic treatment with glucocorticoids B6D2 mice were treated topically with vehicle (control) or glucocorticoid FA (2 μg/animal), every 72 h for 2 wk (3 mice/group). Human volunteers were treated topically on the left arm with 0.05% CBP cream daily for 2 wk. Untreated skin from the right arm was used as control. Mouse skin and whole human skin biopsies were collected 24 h after the indicated treatment. **a.** Total RNA from the epidermis of individual mice treated with FA x1, x2, x3, or x4 times were used for Q-PCR analysis of *Klk6* expression. *Rpl27* was used as a cDNA normalization control. Results are presented as mean ± SD. **p<0.01 vs. control. **b.** Western blot analysis of KLK6 protein from epidermis of individual animals treated with either vehicle (c) or FA x4. Glyceraldehyde-3-phosphate dehydrogenase (GAPDH) was used as loading control. **c.** Induction of *KLK6* mRNA expression in human skin by CBP. Total RNA was extracted from whole skin biopsies and subjected to RT-PCR. *RPL27* was used as a cDNA normalization control. **d.** KLK6 immunostaining of mouse skin treated with vehicle (control) or FA every 72 h four times (FA x4). **e.** Double KLK6 (red) and BrdU (brown) immunostaining of mouse skin treated as in (d). Arrowheads and arrows indicate the expression of KLK6^+^ and BrdU^+^ keratinocytes, respectively. Scale bars, 20 μm (d) and 10 μm (e). SG – sebaceous glands. Inserts, higher magnification of BrdU^+^- and Klk6^+^-keratinocytes; dashed line indicates border between epidermis and dermis. Note: in mouse skin chronically treated with FA, BrdU^+^ keratinocytes in basal layer of interfollicular epidermis were frequently adjacent to KLK6^+^ suprabasal keratinocytes.

It is known that *KLK6* transcription is regulated by steroid hormones [19], but the glucocorticoid effect on KLK6, especially *in vivo*, has not been well studied. We treated F1 C57BL x DBA (B6D2) mice with FA, a medium potency glucocorticoid widely used for the treatment of patients with inflammatory dermatological diseases. We performed Q-RT-PCR using total RNA from mouse epidermis after 1-4 FA applications (2 μg/mouse, applied every 72 h). The level of *Klk6* mRNA was low in vehicle-treated control skin, and relatively weakly induced during the first week of FA treatment (FAx1 and x2), but became strongly elevated after third and fourth FA applications (x3 and x4, Figure 1a). These findings were confirmed by Western blot (Figure 1b) and immunostaining that showed non-detectable KLK6 levels in the IFE of control mice and multiple KLK6-positive suprabasal keratinocytes after long-term FA treatment (Figure 1d). We also observed positive KLK6 staining in the bulge keratinocytes and in sebaceous glands with and without FA treatment, but did not notice any significant KLK6 induction after FA (Figure 1d and Supplemental Figure 1).

Since mouse skin develops resistance to FA anti-proliferative effects by the end of a 2-week-treatment [11], we wanted to determine whether induced KLK6 expression was associated with keratinocyte proliferation. Using double immunostaining for KLK6 and the proliferation marker BrdU, we found that even though KLK6 protein was expressed in non-proliferating suprabasal keratinocytes, KLK6^+^ cells were frequently adjacent to BrdU^+^ cells in the basal layer (Figure 1e). We found previously that most of proliferating keratinocytes in the skin chronically treated with glucocorticoids were localized in the IFE, but not in the bulge stem cell niche [8]. We confirmed this earlier finding here (Figures 1e and 3a). Importantly, the major surge of KLK6 expression was also found in IFE (Figure 1d and Supplemental Figure 1). These interesting observations suggest that there may be a connection between induced KLK6 expression and recovered proliferation of keratinocytes after chronic glucocorticoid applications.

For human volunteers, we used one of the most potent steroids, clobetasol propionate (CBP, 0.05% cream) [5,29]. *KLK6* mRNA expression was strongly induced in human skin after 2 weeks of treatment with CBP (Figure 1c). This clinically relevant regiment of topical treatment induces significant skin atrophy and 50% epidermal thinning [30]. Thus, the KLK6 induction response to atrophogenic regiments of glucocorticoid treatment was similar in human and murine skin.

### Development of glucocorticoid-induced skin atrophy does not depend on KLK6 status

To examine whether induced KLK6 expression is causally linked to development of proliferative resistance to glucocorticoids, we used KLK6 KO mice generated by Dr. Yoshida *et al*. [28]. KLK6 null mice do not have a major phenotype, except the modestly decreased expression of myelin proteins that could result in altered regeneration after spinal cord injury [28]. The skin phenotype in these animals has not been previously studied.

**Figure 2:**
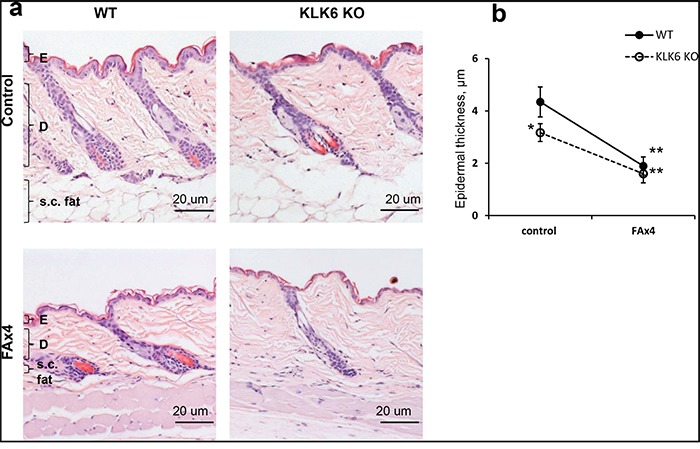
Glucocorticoid-induced skin atrophy in wild type (WT) and KLK6 KO mice KLK6 KO and WT isogenic C57Bl mice were treated topically with vehicle (control) or glucocorticoid FA (3 μg/animal) every 72 h four times (FAx4). Skins were collected 24 h after the last treatment. **a.** H&E staining of WT and KLK6 KO skin. Scale bar, 20 μm. E - epidermis; D - dermis; s.c. fat -subcutaneous fat. **b.** Epidermal thickness of vehicle- and FA-treated WT and KLK6 KO mouse skin was measured as described in Materials and Methods. Mean ± SD (3 mice/group) is presented in μm. *p<0.05 KLK6 KO control vs. WT control; **p<0.001 FAx4 vs. corresponding control.

**Figure 3:**
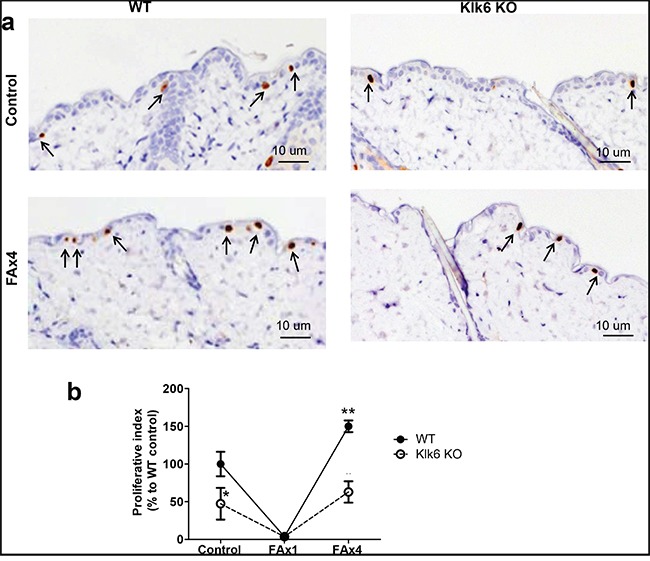
Development of proliferative resistance to chronic glucocorticoid is decreased in skin of KLK6 KO mice Isogenic WT and KLK6 KO mice (3/group) were treated topically with acetone (vehicle control) or glucocorticoid FA (3 μg/animal) once (FAx1) or every 72 h for 2 wk (FAx4). Skins were collected 24 h after the last treatment. **a.** BrdU immunostaining in WT and KLK6 KO skin. Arrows point to the interfollicular BrdU^+^ cells. Scale bar, 10 μm. Note: increased number of BrdU^+^-keratinocytes in chronically FA-treated compared to control-treated skin particularly in WT mice. **b.** Quantification of proliferation in skin. The proliferative index (number of BrdU^+^-basal keratinocytes/total number of basal keratinocytes) was determined as described in Materials and Methods and is presented (mean ± SD) as % relative to the proliferative index in the skin of control WT mice. *p<0.05 KLK6 KO control vs. WT control; **p<0.01 FAx4 vs. corresponding control animals.

We found that KLK6 KO skin was mildly hypoplastic and that KLK6 KO epidermis was ~ 25% thinner than in WT skin (Figure 2b). In addition, the relative number of proliferating BrdU^+^ epidermal basal cells in KLK6 KO was less than 50% of that in WT epidermis (Figure 3b). Thus, a deficiency of KLK6 resulted in reduced keratinocyte proliferation leading to fewer cells and cell layers in the epidermis.

As reported previously [11], chronic FA treatments induced skin atrophy in WT mice with thinning of the epidermis, atrophy of the sebaceous glands and hair follicles, and an almost complete loss of the subcutaneous fat layer (Figure 2a). After chronic FA, KLK6 KO skin looked similar to WT mice with severe atrophy of epidermis, sebaceous glands, hair follicles and subcutaneous fat (Figure 2a). However, since the KLK6 KO epidermis in adult mice was hypoplastic to begin with, the relative reduction of epidermal thickness compared to control was slightly less than in WT mice (Figure 2b). Thus, the lack of KLK6 did not affect the induction of skin atrophy by chronic topical glucocorticoid.

### The development of proliferative resistance to glucocorticoids (tachyphylaxis) is decreased in KLK6 KO skin

The development of resistance to the anti-proliferative effects of glucocorticoids was examined by the numbers of BrdU^+^ basal keratinocytes in the beginning and at the end of chronic FA treatment. Proliferation was almost completely shut down after acute FA application in both genotypes, suggesting that KLK6 is not required for FA growth inhibitory effects (Figure 3b). Chronic FA treatment resulted in a burst of proliferation in WT mice that exceeded the control level by 1.5-fold (Figure 3b). Although there was also a rebound in proliferation in KLK6 KO epidermis, the number of BrdU^+^ cells in the KLK6 KO epidermis was only approaching the relatively low control level and was significantly, 2.5-fold less than that of WT mice (Figure 3b). These findings suggest that the regeneration process is decreased in KLK6 KO skin and that KLK6 induction is at least in part responsible for the development of tachyphylaxis after prolonged steroid treatment.

### GR expression and function are not affected in KLK6 KO skin

Next we assessed whether the delay in tachyphylaxis development in KLK6 KO epidermis was linked to the changes in the expression and function of the GR, as assessed by the induction of several well-known GR target genes that are part of GR signature in mouse and human skin [30]. These included genes encoding cytochrome Cyp2b10, involved in steroid and xenobiotic metabolism; Fkbp51, a molecular chaperone involved in GR activation; and Redd1, a negative regulator of mTOR [30–33].

**Figure 4:**
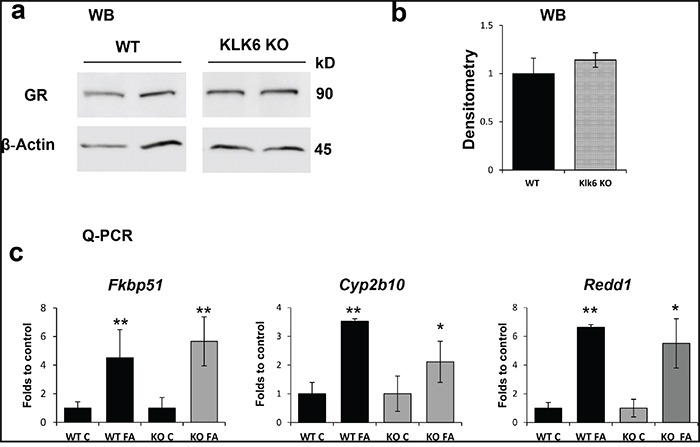
The expression and function of the glucocorticoid receptor is not affected by the lack of KLK6 **a.** Western blot analysis of epidermal proteins from WT and KLK6 KO individual mice (2/group) treated with vehicle (x 24 h). β-actin was used as a loading control. **b.** Quantitative analysis of GR protein expression. GR band densities were measured by Image J software, normalized against β-actin expression and are presented as mean ± SD. **c.** FA-induction of GR target genes *Fkbp51*, *Cyp2b10*, and *Redd1* in the epidermis of WT and KLK6 KO mice. Isogenic WT and KLK6 KO mice (3/group) were treated topically once with acetone (control) or FA (3 μg/animal) and skins were collected 24 h later. Total RNA was isolated from the epidermis and used for Q-PCR as described in Materials and Methods. *Rpl27* was used as a loading/normalization control. Results are presented as mean ± SD. *p<0.05 **p<0.01 vs. corresponding control.

The amount of GR protein is one of the major determinants of cell response to glucocorticoids. We found that the amount of GR protein did not differ in WT and KLK6 KO epidermis (Figure 4a, 4b). The induction of GR target genes was measured 24 h after a single FA application, when GR activation is usually fully developed [29]. The changes in the expression of all genes under study were not statistically different in WT and KLK6 null epidermis (Figure 4c), suggesting that neither GR expression nor GR signaling were significantly affected in KLK6 KO keratinocytes.

## DISCUSSION

Tachyphylaxis is the development of tolerance or drug resistance to a therapeutic agent with continued use and is a major clinical problem when treating many chronic diseases [13,34]. Tachyphylaxis to topical glucocorticoids has been noted for decades in rodent and also human skin, even though there has been a debate regarding the development of steroid resistance in patients. Indeed, some studies suggest that the phenomenon of “steroid resistance” in human skin can reflect the irregular or discontinued use of glucocorticoids by patients during the treatment course [12–14]. Nonetheless, it is well documented that human skin develops resistance to the vasoconstrictive effects and suppression of histamine-induced wheal formation by glucocorticoids after continuous applications [7–9]. We showed previously that it takes approximately two weeks for the development of steroid resistance in mouse interfollicular keratinocytes [11]. Interestingly, the epidermal stem cells in hair follicle bulge niche remain sensitive to chronic glucocorticoids during whole course of treatment [11].

We found that KLK6 was strongly upregulated in atrophic mouse and human skin chronically treated with steroids. Estrogens and progestins, and to a lesser extent androgens, have been shown to activate *KLK6* and other *KLK*s expression [19,35,36]. The limited studies of *KLK6* regulation by glucocorticoids indicated that the glucocorticoid effect was positive or negative depending on cell type [19]. It was also shown that steroid hormones can simultaneously activate several genes from the *KLK* family in breast cancer cells [36]. We found that in mouse skin after chronic glucocorticoid applications, the expression of *Klk10*, *11* and *8* was increased by 1.5-2.0 fold (data not shown), much less compared to ~6-fold *Klk*6 induction (Figure 1a).

Interestingly, the previous bioinformatics analysis of upstream *KLK6* proximal promoter sequences had not identified any putative steroid hormone-response elements [36]. Our analysis of ~1 kb of the human *KLK6* promoter by TFBIND/Transfac (http://tfbind.hgc.jp/; http://www.gene-regulation.com/pub/databases.html) revealed several putative GR binding sites (GRE; data not shown). However, further analysis of GR binding to *KLK6* regulatory sequences by ENCODE (Encyclopedia of DNA Elements https://genome.ucsc.edu/ENCODE/, and ChEA 2015 (in-house ChIP-seq database of mammalian transcription factor interactions with DNA, Ma'ayan and Dudley, Icahn School of Medicine, Mountain Sinai Hospital, NYC, NY) did not confirm that GR binds to these GREs. This most probably reflects the fact that GR and other steroid hormone receptors usually bind to very distant (10-20 Kb from the start codon) regulatory sequences [38]. Additionally, it is known that in many instances GR regulates gene expression via DNA binding-independent mechanisms, for example via interaction with other transcription factors [14, 15].

Previously, we examined gene induction by a non-steroidal compound/selective activator of GR called Compound A. Compound A, in contrast to glucocorticoids, did not induce KLK6 expression (data not shown), which may correlate with both general inability of Compound A to induce activation of GR target genes (Compound A is known to shift GR signaling towards gene transrepression) and its inability to cause skin atrophy [37–39].

Several lines of evidence presented here suggest that KLK6 is indeed involved in normal epidermal maintenance and homeostasis and plays a role in the resistance of keratinocytes to the antiproliferative effects of glucocorticoids, even though it did not significantly affect GR signaling. Untreated KLK6 KO epidermis was hypoplastic with fewer proliferating basal keratinocytes, which was consistent with a previous report that showed ectopically overexpressed KLK6 increased keratinocyte proliferation [21]. This suggests that even a low level of KLK6 expression in untreated skin is necessary for the optimal epidermal homeostasis.

Interestingly, aged skin has a phenotype similar to steroid-induced skin atrophy [40]. It is known that the expression of GR and the expression of the glucocorticoid-activating enzyme, 11β-hydroxysteroid dehydrogenase type 1 (11β-HSD1), are increased in aged skin, and this may contribute significantly to skin aging phenotype [40–42]. As we showed here, KLK6 plays an important role in skin protection against glucocorticoid-induced skin atrophy. Even though the changes in KLK6 expression/activity in aged skin has not been studied, it was shown that the total trypsin-like activity related to the KLK5, 6, and 8 declined in epidermis with age [43]. Thus, it is possible that the natural anti-atrophogenic signaling mediated by KLK6 is diminished in the skin of elderly people. However, at this time there is no evidence for cross-talk between GR and KLK6 in aged skin.

Even though KLK6 was dispensable for keratinocyte growth inhibition by glucocorticoids at the beginning of treatment, it played an important role in the development of proliferative resistance to these hormones and keratinocytes lacking KLK6 were less able to recover from the growth blockade by FA. In line with our previous observations [8], the overall burst of proliferation during tachyphylaxis occurred in IFE, and this coincided with the prevalent KLK6 expression in the same epidermal compartment. Remarkably, KLK6 expression was frequently seen in suprabasal keratinocytes adjacent to FA-resistant proliferating basal keratinocytes. Mechanistically, how a secreted proteinase such as KLK6 transduces a proliferative signal to basal keratinocytes has yet to be determined. One possibility is that KLK6 could be involved in a proteolytic signaling cascade that ends in cleavage and release of ligand(s) for growth factor receptors stimulating proliferation and/or cleavage of cell adhesion molecules, allowing for migration and proliferation of keratinocytes. In addition, KLK6 is also known to target extracellular matrix and basement membrane proteins, including fibronectin, laminin, vitronectin and collagen, specifically several subunits of type IV collagen (http://www.genecards.org/, Keggs pathways). Thus, it is possible that KLK6 could affect keratinocyte behavior via its effects on the basement membrane that plays a central role in keratinocyte proliferation and migration as well as in differentiation of epidermis.

Our results are consistent with reports regarding the increased KLK6 expression in several proliferative skin diseases such as psoriasis and atopic dermatitis [24,26,27], as well as in human premalignant skin lesions and squamous cell carcinomas [21]. Interestingly, the KLK6 expression is also strongly induced in epidermis after skin wounding [44], and skin wound healing is accelerated in KLK6 transgenic mice possibly due to increased keratinocyte proliferation and motility [21]. Even though the mechanisms of skin regeneration after steroid-induced atrophy are not well known, the induction of KLK6 seems to be common for both wound healing and the regeneration after steroids.

Interestingly, we recently found that at least two early genes activated by glucocorticoids in WT skin within hours after acute treatment and elevated during the whole treatment cycle, *Fkbp51* and *Redd1*, act as atrophogenes in skin and are causatively involved in skin hypoplasia induced by steroids [30,33]. The role of those persistently activated genes is in striking contrast to the effect of KLK6 in skin where it acts as skin protector involved in the development of proliferative resistance to steroids. Overall, our results suggest a novel mechanism of epidermal regeneration after glucocorticoid-induced atrophy via KLK6 activation.

## MATERIALS AND METHODS

### Chemicals

Fluocinolone acetonide (FA), BrdU and all other chemicals, unless stated otherwise, were purchased from Sigma. Clobetasol propionate (CBP) was purchased at the pharmacy as 0.05% cream.

### Animals and treatments

B6D2 (F1 C57BL/6 х DBA) females were from Jackson Laboratory (Bar Harbor, ME). KLK6 KO mice on C57BL/6 genetic background were described previously [28]. Isogenic WT C57BL/6 mice were from Sankyo Labo Service Corporation (Japan). Six-seven wk old females in the telogen stage of the hair cycle were shaved and treated 3 days later. FA was applied topically (2 μg in B6D2, and 3 μg in C57BL/6 and KLK6 KO mice) in 200 μl acetone to the back skin once or four times (every 72 h) during two weeks as described previously [11]. Control animals were treated with acetone only. Skin was harvested 24 h after a single or last FA application (during chronic treatment) as indicated in the Figure legends. Where indicated, mice were injected intraperitoneally with BrdU (50 μg/g body weight) 1 h before sacrifice. The experiments with WT isogenic animals were repeated three times, and with KLK6 KO animals were repeated twice. All animal experiments were approved by the Asahikawa Medical University and Northwestern University Animal Care and Use Committees.

### Human volunteer treatment

The glucocorticoid CBP was applied topically as a 0.05% cream to the skin of right arm of healthy human volunteers (age 32-65) every 24 h for two wk. Untreated left arm skin was used as control. Five mm punch skin biopsies were taken 24 h after last CBP application and used for RNA extraction.

All human studies were approved by Northwestern University Institutional Review Board. Written informed consent was received from the volunteers before participation.

### Western blot analysis

Epidermis was isolated from dermis by scraping [45–47] and homogenized in RIPA buffer with protease inhibitor cocktail (Thermo Scientific, Waltham, MA). The purity of epidermis isolation by this approach was verified previously by us using specific keratinocyte and other skin cells markers [48]. The extracted whole cell proteins were subjected to SDS-PAGE and transferred to nitrocellulose membranes (BioRad, Hercules, CA). Membranes were incubated with primary antibody overnight at 4°C, followed by peroxidase-conjugated anti-rabbit IgG secondary antibodies (Cell Signaling Technology, Beverly, MA). Protein bands were visualized using ECL detection kit (Amersham Bioscience, Uppsala, Sweden) and analyzed by Image Analyzer LAS-3000 (Fuji, Tokyo, Japan). The antibodies are listed in the Supplemental Table S2.

### Histological analysis and immunostaining

Formalin-fixed, paraffin-embedded skin sections were stained with hematoxylin and eosin (H&E) or with primary antibodies against BrdU and mouse KLK6 at 4°C overnight followed by anti-mouse horseradish peroxidase- and anti-rabbit alkaline phosphatase-linked secondary antibodies from MACH 2 Double Stain 2 (Biocare Medical, Concord, CA). Immunoperoxidase reaction was visualized using the Dako Envision system plus HRP/DAB Kit (Dako, Tokyo, Japan) and alkaline phosphatase activity was detected by Vulcan Fast Red Chromogen Kit 2 (Biocare Medical, Concord, CA).

### Morphometric analysis

Quantification of the epidermal width (as the readout for skin thinning) was performed in H&E skin sections using the Image-Pro PLUS software (Media Cybernetics, Bethesda, MD). To assess keratinocyte proliferation, the number of proliferating (BrdU^+^) and total basal keratinocytes was counted, and proliferative index (number of BrdU^+^ basal keratinocytes/total number of basal keratinocytes) is presented as % relative to the proliferative index in WT control epidermis. For all morphometric studies, 20 individual fields of view/slide in three individual mouse skin samples/treatment (at least 60 images/treatment group) were analyzed.

### RNA preparation and PCR analysis

After euthanasia, dorsal skins of mice were removed, and epidermis was mechanically separated by scraping as described [11,47]. Total RNA from mouse epidermis or whole-thickness human skin biopsies was isolated according to the protocol for RNeasy Fibrous Tissue Mini Kit (Qiagen, Germantown, MD). RNA was reverse transcribed with iScript™ cDNA Synthesis Kit (Bio-Rad, Hercules, CA), and used for quantitative Q-PCR or semi-quantitative two-step RT-PCR. The gene-specific primers were designed with NCBI Primer-BLAST (Supplemental Table S1). Ribosomal protein L27 (*Rpl27*) RNA was used as a normalization control [48, 49]. Both RT-PCR and Q-PCR were performed using individual RNA samples from volunteers and mice (3 samples/experimental group).

### Statistical analysis

Data are presented as mean ± SD. The two-tailed Student's t-test was applied to analyze the differences between two groups. *P* < 0.05 was considered to be statistically significant. All experiments were repeated two to three times. In animal experiments we used at least three animals/group. In all figures the results of one representative experiment are shown.

## SUPPLEMENTARY DATA



## Abbreviations

11 β-HSD1: 11 β-hydroxysteroid dehydrogenase
BrdU: 5-bromo-2'-deoxyuridine
CBP: clobetasol propionate
FA: fluocinolone acetonide
GR: glucocorticoid receptor
GRE: glucocorticoid responsive element
H&E: hematoxylin and eosin
IFE: interfollicular epidermis
KLK6: kallikrein-related peptidase 6
KO: knockout
Rpl27: ribosomal protein L27
RT-PCR: reverse-transcribed polymerase chain reaction.
