# Supplementary material for: Important role of kallikrein 6 for the development of keratinocyte proliferative resistance to topical glucocorticoids
Source: Oncotarget. 2016 Jun 8;7(43):69479–88. doi: 10.18632/oncotarget.9926 (PMC5342492; doi:10.18632/oncotarget.9926)
Supplement: Supplementary file 1 [file oncotarget-07-69479-s001.pdf]

Important role of kallikrein 6 for the development of keratinocyte proliferative resistance to topical glucocorticoids

SUPPLEMENTARY DATA

SUPPLEMENTARY REFERENCE

Terayama R, Bando Y, Jiang YP, et al. (2005). Differential expression of protease M/neurosin in oligodendrocytes and their progenitors in an animal model of multiple sclerosis. Neurosci Lett 382: 82-7.

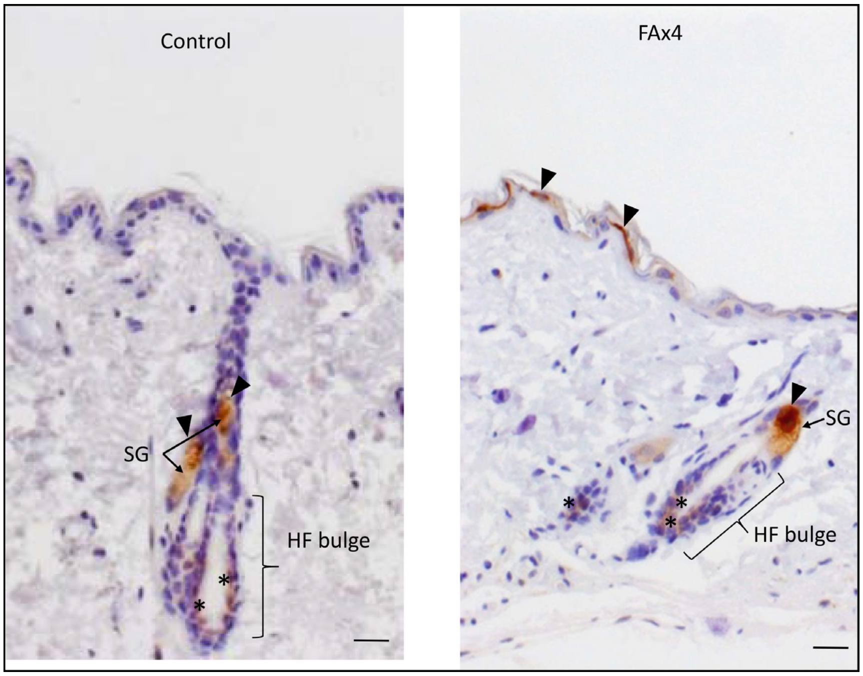

Supplementary Figure S1: KLK6 immunostaining of mouse skin. B6D2 mice were treated topically with vehicle (control) or glucocorticoid FA, every 72 h for 2 wks (FAx4). Mouse skins were collected 24 h after the indicated treatment. Arrowheads and stars indicate KLK+ cells in sebaceous glands (SG), HF bulge, and interfollicular epidermis. Scale bars, 10 µm.

Supplementary Table S1: Summary of the primers used in the study

Gene symbol		Primer sequence: sense/antisense (5'-3')
**mouse**	**human**	
Klk6		GCT GGG GCA AGA TGG AAA ATG GTG A ACA GGG<br>CAT GTC ACC CCA TGA CA
Redd1		GGG CCG GAG GAA GAC TCC TCA TA<br>CTG TAT GCC AGG CGC AGG AGT TC
Fkbp51		GGT TTT GGA GAA GCC GGG AAG CC<br>CCT GCG TGT ACT TGC CTC CCT TG
Cyp2b10		CCA ACC TTC AAG GAA TAT GGT GTG<br>GCA GAT AAT ATT GGC CGT GAT GC
Rpl27		GCC CTG GTG GCT GGA ATT GAC C<br>TTG CGC TTC AAA GCT GGG TCC C
	KLK6	CAC CAC CAG CTG CCA CAT CCT G<br>CAC ATA CCA GCG GAC CCC CAG A
	RPL27	ACC GCT ACC CCC GCA AAG TG<br>CCC GTC GGG CCT TGC GTT TA

Supplementary Table S2: Summary of the antibodies used in the study

Antibody	Assay	Dilution	Company
anti-mouse Klk6	IHC and WB	1:1000	Terayama et al., 2005
anti-human KLK6	IHC	1:100	Abcam, Cambridge, UK
anti-BrdU	IHC	1:1000	BD Pharmingen, San Jose, CA
anti-GR	WB	1:1000	Santa Cruz Biotechnology, Pasadena, CA
anti-Actin	WB	1:1000	Santa Cruz Biotechnology, Pasadena, CA

IHC: immunohistochemistry, WB: Western Blot
